# Sterilized Lint

**Published:** 1890-02

**Authors:** 


STERILIZED LINT.—M. Regnier renders lint sterile by heating it to a temperature of 120° C. (248° F.). M. Regnier has tested the antiseptic value of lint thus prepared in dressings applied after operations of various kinds with good results. At the recent Surgical Congress he stated that he considered sterilized lint equal to antiseptic dressings.—*N. Y. Med. Record.*

